# A Pragmatic Outpatient Framework for Appetite‐Related Phenotyping in Obesity

**DOI:** 10.1002/osp4.70184

**Published:** 2026-08-07

**Authors:** Jassel Arturo Velázquez‐Navarro, Roberto Rodríguez‐Moriel, Roxana Michel Márquez‐Herrera, Luis Antonio Ramos‐Márquez

**Affiliations:** ^1^ Department of Endocrinology Angeles Hospital Chihuahua Mexico; ^2^ Department of Endocrinology Christus Muguerza Hospital del Parque Chihuahua Mexico; ^3^ Center for Obesity Diabetes and Technology Mexico‐American Hospital Guadalajara Jalisco Mexico; ^4^ Department of Food and Nutrition University of Guadalajara Guadalajara Jalisco México

**Keywords:** emotional eating, obesity, phenotype, precision medicine, satiety

## Abstract

**Background:**

Previous obesity phenotyping models have been proposed; however, current protocols rely on complex physiological assessments that are difficult to implement in routine outpatient care.

**Aim:**

To describe and apply a pragmatic outpatient framework for appetite‐related phenotyping integrating standardized satiety testing and emotional eating assessment.

**Methods:**

In this observational study, adults with overweight or obesity underwent a standardized mixed‐meal satiety test using visual analogue scales together with emotional eating assessment using the Emotional Eating Questionnaire. Exploratory thresholds were derived from a normal‐weight reference group. Participants were classified into appetite‐related phenotypes: impaired early satiety, impaired late satiety, emotional eating, overlap, and no dominant phenotype.

**Results:**

A total of 129 adults with overweight or obesity were included. Impaired early satiety was identified in 48.1% of participants, impaired late satiety in 35.7%, and normal satiety responses in 16.3%. Severe emotional eating was present in 12.4% of participants. Overlap between emotional eating and altered satiety‐test responses was identified in 11% of participants.

**Conclusions:**

Most participants exhibited at least one appetite‐related phenotype, supporting the relevance of assessing both homeostatic (satiety) and emotional dimensions of eating behavior in clinical practice. Future studies should evaluate reproducibility and potential implications for phenotype‐guided obesity treatment.

## 1 Introduction

Obesity is a chronic, relapsing disease characterized by considerable heterogeneity in the mechanisms contributing to weight gain and weight regulation [1, 2]. Current treatment strategies remain largely guided by body mass index (BMI) thresholds and obesity‐related complications [3]. However, although these are useful for estimating cardiometabolic risk and determining treatment eligibility, they do not adequately capture interindividual differences in appetite regulation or eating behavior.

Increasing evidence supports the concept of obesity as a disorder of impaired energy homeostasis involving dysregulation of central appetite and reward pathways together with altered gastrointestinal satiety signaling [4, 5, 6, 7, 8, 9]. These mechanisms may interact differently across individuals, potentially contributing to variability in clinical presentation and pharmacological treatment response observed among patients with similar BMI values, including the well‐recognized phenomenon of partial responders and non‐responders to anti‐obesity medications [10, 11].

Previous phenotype‐based models have identified clinically actionable subgroups related to abnormal satiation, impaired postprandial satiety, hedonic eating behavior, and reduced energy expenditure, suggesting that recognition of dominant obesity‐related mechanisms may help guide therapeutic decision‐making [12, 13]. This has become increasingly relevant with the rapid expansion of anti‐obesity pharmacotherapy, particularly in the era of incretin‐based therapies [14, 15]. These advances have increased interest in phenotype‐guided treatment selection in routine outpatient care [16].

Among appetite‐regulating mechanisms, it is important to distinguish satiation, the processes that develop during a meal and determine meal size, from satiety, the postprandial inhibition of hunger that delays the next eating episode [17, 18]. Because satiety itself evolves over the postprandial period [18, 19, 20], an early meal‐proximate phase can be operationally distinguished from a later phase in which fullness is sustained before hunger returns. Because different stages of this cascade may be impaired independently, distinguishing them may help capture distinct points of failure with implications for phenotype‐based therapeutic selection.

However, most appetite phenotyping strategies currently rely on specialized physiological assessments such as ad libitum meal testing, gastric emptying, and extensive behavioral testing [12, 19]. While these methods provide relevant mechanistic information, their complexity and resource requirements limit their use in routine outpatient care. As a result, translation of appetite phenotyping into routine clinical practice remains limited.

In contrast, standardized appetite assessment tools, particularly visual analogue scales (VAS) and validated eating‐behavior questionnaires, have been extensively used in human appetite research and may facilitate recognition of appetite‐related alterations in routine obesity care [18].

Therefore, the aim of the present study was to describe and apply a pragmatic approach for appetite‐related phenotyping in obesity by integrating standardized VAS and the Emotional Eating Questionnaire (EEQ). By combining clinically interpretable satiety testing with behavioral assessment in a real‐world setting, this study provides an initial step toward translating obesity phenotyping into clinical practice.

## 2 Materials and Methods

### 2.1 Study Design and Participants

This observational study included two groups consecutively recruited from an outpatient endocrinology clinic. A normal‐weight reference group underwent a standardized mixed‐meal satiety test under controlled outpatient conditions to establish a reference distribution of postprandial appetite responses. A second group of adults with overweight or obesity underwent the same standardized satiety test and was subsequently classified into appetite‐related phenotypes according to predefined thresholds derived from the reference group.

The reference group consisted of adults with normal body weight (BMI 18.5–24.9 kg/m^2^) without known metabolic disease.

The overweight/obesity group included adults with BMI ≥ 27 kg/m^2^ with or without adiposity‐related comorbidities evaluated in routine outpatient obesity care. Eligible participants were ≥ 18 years of age and completed both the EEQ and the standardized satiety test. Exclusion criteria in both groups comprised factors and treatments known to alter appetite, satiety response, or weight regulation, —namely diabetes mellitus, previous bariatric surgery, current anti‐obesity pharmacotherapy, and current use of antidepressant or anxiolytic medications—to limit confounding of the appetite‐phenotype assessment.

### 2.2 Standardized Appetite Assessment Protocol

Satiety‐related responses were assessed using a standardized mixed‐meal satiety test performed in outpatient conditions. Subjective appetite perceptions were evaluated using 100‐mm VAS, widely used in human appetite research to assess hunger and satiety sensations [18].

Participants underwent testing in the morning after an overnight fast of at least 8 hours and were instructed to avoid caffeine, alcohol, nicotine exposure, and vigorous physical activity prior to testing to minimize variability during appetite assessment. Participants were instructed not to initiate the test unless baseline hunger exceeded 50 mm on the VAS to reduce variability associated with heterogeneous baseline appetite states [18, 20].

The standardized mixed‐meal consisted of a sandwich (two slices of whole‐grain bread and three slices of ham) together with 237 mL of Ensure Advance, yielding approximately 600 kcal, with 51% of the total caloric content derived from carbohydrates, 19% from protein, and 30% from fat, to provide a standardized satiety stimulus while preserving clinical feasibility [18, 20, 21, 22, 23, 24]. Participants were instructed to consume the meal within 15 minutes. Hunger and satiety were assessed at the fasting baseline, 30 minutes after meal initiation, and 120 minutes postprandially using 100‐mm VAS. The 30‐min satiety measurement was used as the immediate postprandial satiety score, while the change between baseline and 30‐min satiety ratings was used to calculate Δ satiety. Hunger at 120 minutes postprandially was used as the measure of postprandial hunger recurrence. These time points were selected to capture both the early satiety response following meal ingestion and the subsequent return of hunger during the postprandial period, consistent with prior studies [18, 20, 25].

### 2.3 Exploratory Reference‐Distribution Thresholds

Results from the standardized satiety test in the normal‐weight reference group were used to establish a reference distribution of postprandial subjective appetite responses under standardized conditions [26, 27]. Because universally accepted clinical thresholds for VAS‐derived satiety responses in obesity phenotyping are currently lacking, exploratory thresholds were derived from the tails of the observed response distribution in the normal‐weight group (corresponding to the percentile 5 and 95 values) and subsequently applied to classify appetite‐related phenotypes in the overweight/obesity group. This was intended to support clinically interpretable and feasible appetite phenotyping under routine outpatient conditions. Detailed reference group distributions are provided as Supporting Information S1.

### 2.4 Emotional Eating Assessment

Emotional eating behavior was assessed using the validated EEQ [28]. The questionnaire consists of 10 items evaluating eating behavior in response to emotional and environmental triggers, with total scores ranging from 0 to 30. In accordance with the original EEQ classification, scores were categorized as absent or low emotional eating (0–10), moderate emotional eating (11–20), and very/severe emotional eating (21–30). Participants in the overweight/obesity group completed both the EEQ and the standardized mixed‐meal satiety test.

### 2.5 Appetite‐Related Phenotype Classification

Using predefined thresholds derived from the normal‐weight reference group together with emotional eating assessment, five operational appetite‐related phenotype categories were defined:

- Impaired early‐satiety phenotype: Immediate postprandial satiety < 70 mm on the 100‐mm VAS at 30 minutes and/or Δ Satiety (baseline to 30 minutes) < 35 mm, reflecting a blunted early post‐ingestive satiety response.
- Impaired late‐satiety phenotype: Hunger > 50 mm on the 100‐mm VAS at 120 minutes postprandially, reflecting early recurrence of hunger and poorly sustained late post‐ingestive satiety. Participants meeting criteria for impaired early‐satiety were not additionally classified as having impaired late‐satiety in order to assign each participant to the earliest post‐ingestive satiety defect.
- Emotional‐eating phenotype: A EEQ score ≥ 21 (severe emotional eating) per the original EEQ classification.
- Overlap phenotype: Participants simultaneously met criteria for emotional eating and either impaired early or impaired late satiety.
- No dominant phenotype: An EEQ < 21 and a normal satiety‐test response (absence of impaired early and impaired late satiety).

### 2.6 Ethical Considerations

This study was conducted according to the guidelines laid down in the Declaration of Helsinki and all procedures involving research study participants were approved by the Local Committee of Research and Ethics (No. 017‐2026). Written informed consent was obtained from all subjects/patients.

### 2.7 Statistical Analysis

Continuous variables are presented as median (IQR), and categorical variables as frequencies and percentages. The distribution of emotional eating severity categories according to satiety‐test classification was explored using *χ* ^2^ or Fisher's exact test, as appropriate. All analyses were conducted using Jamovi software, and a two‐sided *p* value < 0.05 was considered statistically significant.

## 3 Results

A total of 129 participants with overweight/obesity were included, of whom 105 (81.4%) were women and 24 (18.6%) were men, age 41 (33–52) years, and BMI 32.3 (29.7–35.3) kg/m^2^.

Impaired early satiety was identified in 62 patients (48.1%), while impaired late satiety was observed in 46 patients (35.7%). The remaining 21 patients (16.3%) were classified as having a normal satiety response.

According to the EEQ, some degree of emotional eating was identified in 116 participants (90%). Mild emotional eating was observed in 40 patients (31%), while the majority exhibited moderate emotional eating (*n* = 60, 46.5%). Severe emotional eating was present in 16 participants (12.4%), which also corresponded to the emotional eating phenotype according to our predefined criterion (EEQ ≥ 21).

**FIGURE 1:**
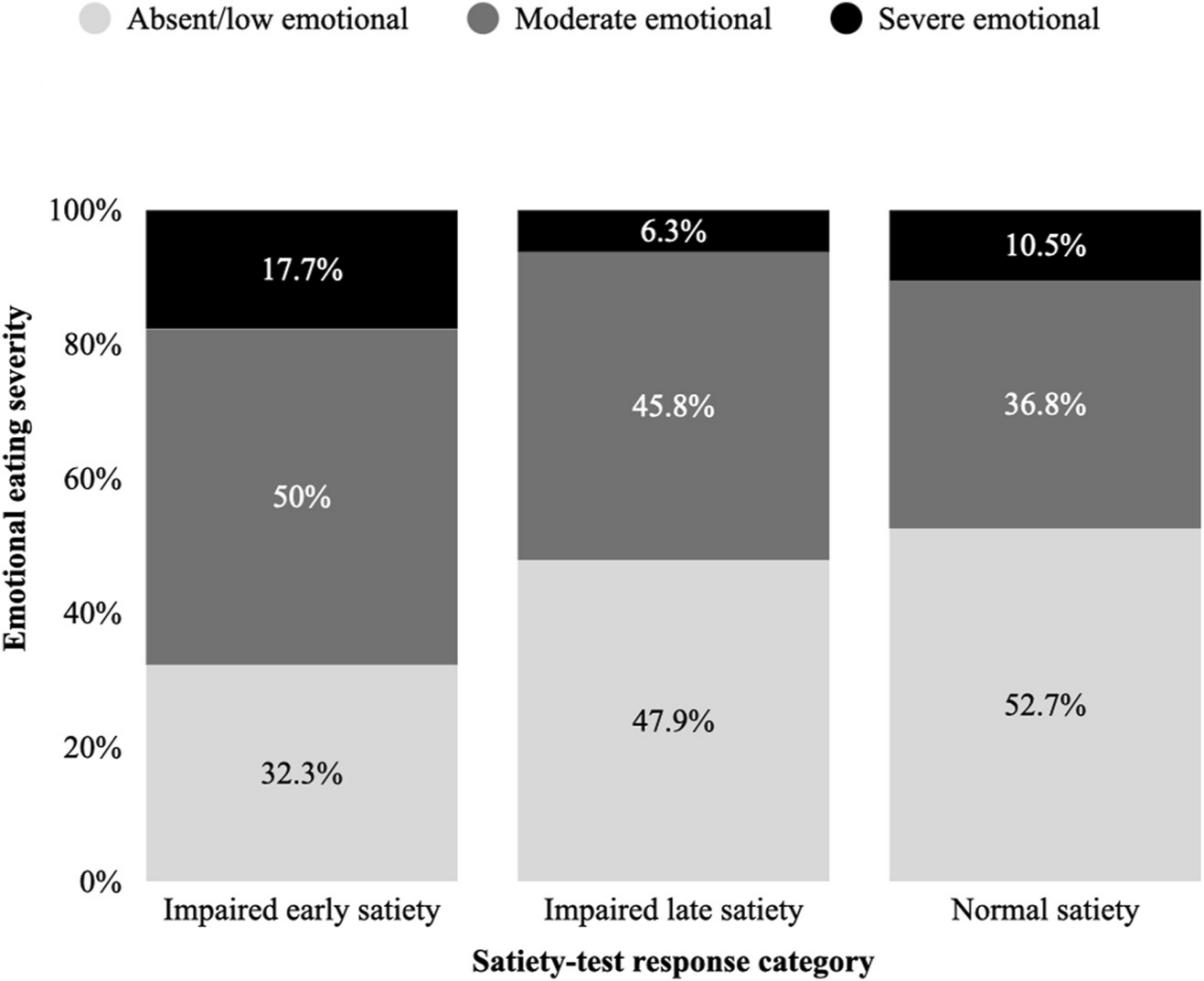
Distribution of emotional eating severity according to satiety‐test classification.

Coexistence between emotional eating and appetite‐related alterations identified through the standardized satiety test was also observed. Figure 1 illustrates the distribution of emotional eating severity categories according to satiety‐test classification. Moderate emotional eating was the predominant category across all satiety‐test groups and was more frequently observed among participants with impaired early satiety and impaired late satiety. Severe emotional eating was also numerically more frequent among participants with impaired early satiety compared with those exhibiting impaired late satiety or a normal satiety‐test profile. However, the overall distribution of emotional eating categories did not differ significantly across satiety‐test classifications (*χ* ^2^ = 6.65, *p* = 0.354).

**FIGURE 2:**
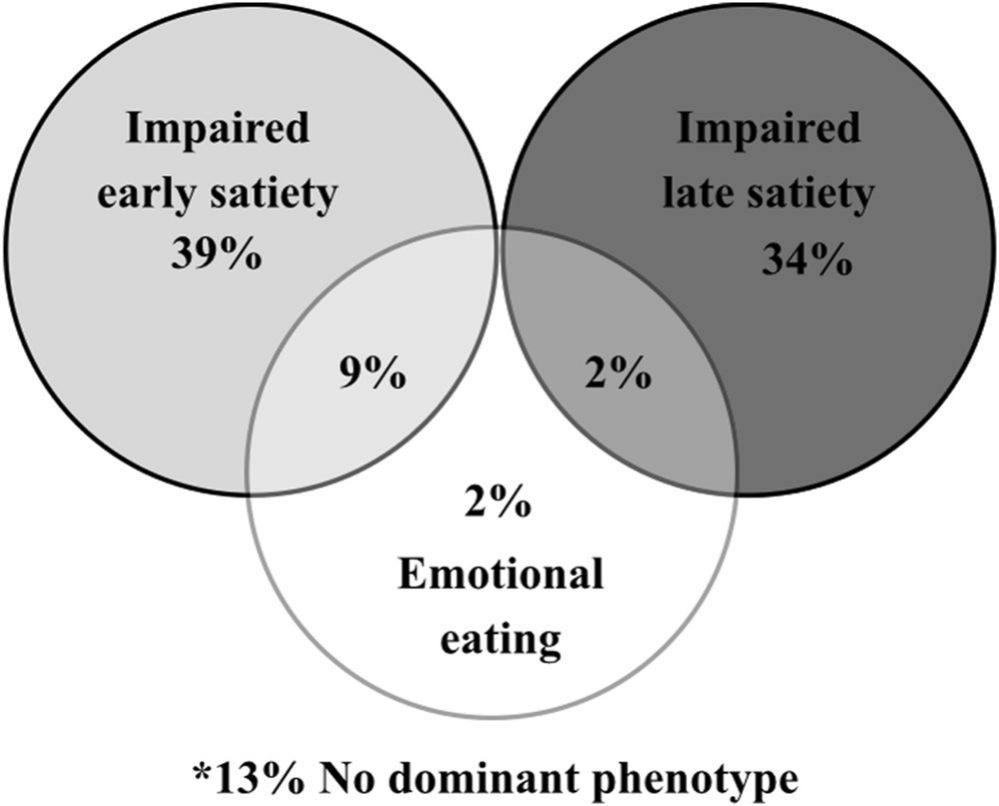
Distribution and overlap of appetite‐related phenotypes within the study population.

Figure 2 illustrates the distribution and overlap of appetite‐related phenotypes within the study population. Impaired early satiety represented the most frequent isolated phenotype, followed by impaired late satiety, whereas isolated emotional eating was uncommon. Overlap phenotypes characterized by the coexistence of emotional eating with a homeostatic alteration were observed more frequently in participants with impaired early satiety than in those with impaired late satiety. As impaired early satiety and impaired late satiety were defined as mutually exclusive categories, no overlap between these two homeostatic phenotypes was identified. Approximately 1 in 8 participants were not classified as having a dominant appetite‐related phenotype.

## 4 Discussion

This study describes a pragmatic outpatient framework for appetite‐related phenotyping in adults with overweight or obesity, integrating standardized VAS‐based satiety testing with emotional eating assessment. The main finding was that most participants exhibited at least one appetite‐related alteration, with impaired early satiety being the most frequent satiety‐test pattern, followed by impaired late satiety, whereas severe emotional eating phenotype was less frequent. These findings highlight the relevance of assessing both homeostatic and emotional dimensions of appetite regulation in routine obesity care [16, 29].

The predominance of impaired early satiety is clinically relevant, since early and late satiety may fail independently and reflect distinct points of failure in the satiety cascade. A patient who fails to feel full soon after eating and one who feels full but rehungers quickly may differ in their dominant mechanism and, potentially, in their response to treatment [17, 18].

Previous phenotype‐based models have identified actionable subgroups of obesity. In the pragmatic trial by Acosta et al. [12], phenotypes were identified in 85% of participants with obesity, two or more phenotypes were present in 27%, and phenotype‐guided pharmacotherapy was associated with greater weight loss than non‐phenotype‐guided treatment. Because, our phenotypes derive from simple, subjective outpatient measures, they should be regarded as operationally distinct from phenotypes defined by specialized procedures such as ad libitum meal testing, gastric‐emptying studies, or calorimetry; the early‐ and late‐satiety phenotypes proposed here may not correspond one‐to‐one to previously described laboratory phenotypes. Instead, our results agree in principle with prior work while testing simpler tools for routine care [13, 16], which is a reason for—rather than against—their independent validation.

An important observation was the co‐existence of emotional eating with altered responses on the standardized satiety test. This overlap suggests that emotional (behaviorally driven) and homeostatic influences on food intake may coexist within the same individual. This is consistent with current models of appetite regulation, in which homeostatic brain–gut signaling and reward‐related pathways interact to influence eating behavior [30]. In contrast, the EEQ adds an accessible behavioral dimension to the subjective satiety information obtained from the standardized test [28]. However, it is important to clarify that emotional eating, as assessed with the EEQ, should not be considered equivalent to food hedonics, which refers specifically to liking and wanting and requires dedicated measures not used here [31].

The group without a dominant appetite‐related phenotype is also informative. In these individuals, obesity may be influenced by mechanisms not captured by the current appetite‐focused model, including reduced energy expenditure, physical activity patterns, sleep, environmental factors, medication exposure, or other biological and behavioral contributors [12, 13].

These findings may have therapeutic implications but should be interpreted cautiously. Current obesity treatment remains strongly guided by BMI thresholds, obesity‐related complications, contraindications, tolerability, cost, and patient preference. At the same time, response to anti‐obesity pharmacotherapy remains heterogeneous, and early non‐response is still commonly identified after treatment initiation rather than predicted beforehand [10, 19].

Phenotype‐guided obesity management has been proposed to align therapy with dominant appetite‐related mechanisms. Impaired early satiety, which may overlap mechanistically with satiation, could respond better to satiety‐enhancing agents such as phentermine/topiramate and also GLP‐1ra; impaired late satiety may respond better to therapies that prolong postprandial fullness, such as GLP‐1ra and GLP‐1/GIP dual agonists [14]. Phenotypes could also guide treatment intensity: earlier use of stronger satiety‐enhancing agents for marked impairment, combination therapy for overlapping phenotypes, and decisions to continue or switch treatment based on early response. In patients without a dominant appetite‐related phenotype, where reduced energy expenditure may play a larger role, emerging therapies targeting energy balance, such as the triple agonist retatrutide, may be relevant [32]. These mechanistic mappings remain hypotheses requiring prospective validation, since the tools used here have not been evaluated as predictors of treatment response.

Beyond selecting anti‐obesity medications according to phenotype, as proposed by Acosta et al. [12], phenotype‐guided care could influence several other aspects of routine treatment. The behavioral intervention could be tailored to the predominant pattern; for example, patients with emotional eating may benefit from structured cognitive‐behavioral or emotion‐regulation strategies rather than pharmacotherapy alone.

These applications, however, center on individual‐level traits and do not capture the broader environment in which eating occurs. Modern food environments, characterized by easy access, low cost, large portion sizes, and aggressive marketing of energy‐dense, highly palatable, ultra‐processed foods, can promote passive overconsumption independently of internal appetite signals [33, 34]. These exposures may increase food intake even in individuals without a predominant appetite phenotype and may further amplify susceptibility in those with impaired satiety or emotional eating. Appetite phenotyping should therefore be viewed as one element within a broader biopsychosocial and environmental model of obesity. Future versions of this framework could also incorporate brief measures of responsiveness to external food cues.

This study proposes a practical phenotyping strategy based on a standardized mixed‐meal challenge, predefined assessment time points, VAS ratings, and a brief validated questionnaire, with limited demands on time and resources [18, 20, 28]. Unlike more complex protocols that require specialized procedures, including ad libitum meal testing, gastric emptying studies, indirect calorimetry, and extensive behavioral assessments [12, 13], this approach may be easier to implement in routine outpatient care. Although comprehensive phenotyping protocols often involve repeated measurements over several hours, a standardized 2‐h evaluation of hunger may provide a simpler alternative while preserving the distinction between early and late satiety and the specific assessment methods required for each [18, 25, 29, 35]. Previous methodological studies have also supported the use of standardized VAS for appetite assessment under controlled conditions [18], reinforcing the clinical and methodological rationale for this approach.

Strengths of this study include its real‐world outpatient setting, the integration of standardized subjective satiety testing, and simple tools implemented without specialized equipment. We did not perform physiological measurements (e.g., gastric emptying, calorimetry, gut‐hormone assays); the test provides standardized appetite ratings in response to a fixed meal challenge rather than a direct physiological readout.

In addition, using a normal‐weight reference group to derive exploratory thresholds under the same testing conditions supports the clinical rationale of the proposed thresholds, although these thresholds should not be interpreted as diagnostic cutoffs or medical decision limits.

Some limitations should be considered. First, the sample was moderate in size and certain population characteristics limited generalizability. The study included relatively few individuals with class III obesity and excluded patients using antidepressant or anxiolytic medications. Because both obesity severity and these medications can influence appetite [36], the phenotype distribution in individuals with these characteristics may differ from that observed in our sample, and appetite phenotyping in this population warrants a dedicated study. Second, although the selected measures are supported by prior physiological and methodological literature, both the proposed classification thresholds and the overall protocol require further validation in independent and more diverse populations. Finally, the present study was designed to assess selected appetite‐related mechanisms and did not capture the full spectrum of obesity phenotypes described in the literature, including impaired satiation and reduced energy expenditure, grazing behaviors, and other biological, psychosocial, or environmental contributors that may influence obesity heterogeneity and treatment response.

In conclusion, this study provides an initial outpatient approach to appetite‐related phenotyping in routine outpatient obesity care. By integrating standardized satiety testing with emotional eating assessment, this offers a simplified, low‐cost, and clinically interpretable approach to identifying dominant appetite‐related patterns. Future studies should validate these operational phenotypes, and determine whether they predict response to lifestyle, behavioral, pharmacological, or combined obesity treatments.

## Author Contributions

J.A.V.N. conceptualized the study, designed the clinical phenotyping protocol, coordinated patient recruitment, collected the data, performed the statistical analyses, interpreted the findings, and drafted the initial manuscript. R.R.M. contributed to study design, patient recruitment, collected the data, and critical revision of the manuscript for important intellectual content. R.M.M.H. contributed to methodological refinement, performed the statistical analyses, and substantial scientific editing of the manuscript. L.A.R.M. contributed to the methodological strategy, clinical and therapeutic interpretation of the findings, and critically reviewed the manuscript for intellectual content. All authors reviewed and approved the final version of the manuscript.

## Funding

The authors have nothing to report.

## Conflicts of Interest

The authors declare no conflicts of interest.

## Supporting information


Supporting Information S1

## Data Availability

The data that support the findings of this study are available on request from the corresponding author. The data are not publicly available due to privacy or ethical restrictions.
