# Supplementary material for: A Pragmatic Outpatient Framework for Appetite‐Related Phenotyping in Obesity
Source: Obes Sci Pract. 2026 Aug 7;12(4):e70184. doi: 10.1002/osp4.70184 (PMC13449295; doi:10.1002/osp4.70184)
Supplement: Supplementary file 1 [file OSP4-12-e70184-s001.docx]

**Supplementary material**

Reference distribution of postprandial appetite responses in the normal-weight cohort used for exploratory phenotype threshold derivation

| **Variable** | **P5** | **P25** | **P50** | **P75** | **P95** |
| --- | --- | --- | --- | --- | --- |
| Postprandial satiety, *VAS mm* | 68 | 78 | 85 | 96 | 100 |
| Δ satiety, *VAS mm* | 33 | 45 | 60 | 70 | 90 |
| 2-h hunger, *VAS mm* | 10 | 20 | 30 | 40 | 52 |

Exploratory operational thresholds for phenotype classification were derived from the tails of the observed response distribution (P5/P95).
